# Global recommendations on adhesion prophylaxis in gynaecological laparoscopic surgery

**DOI:** 10.52054/FVVO.16.3.037

**Published:** 2024-09-30

**Authors:** RL De Wilde, A Adlan, P Aquino, S Becker, M Bigozzi, U Catena, J Clark, F Darmawan, J Dubuisson, MA Habana, CK Khoo, PR Koninckx, H Krentel, A Lam, R Lasmar, SM Mansuria, S Mukherjee, O Musigavong, S Ohri, G Pados, MA Pinho de Oliveira, S Puntambekar, B Rabischong, E Saridogan, J Sehouli, F Sendag, R Paz Tan, V Tanos, R ten Broek, V Tica, LA Torres-de la Roche, M Wallwiener, L Zhu, R Devassy

**Affiliations:** Carl von Ossietzky University, Oldenburg, 26121,Germany; University of Malaysia, Kuala Lumpur, 50603, Malaysia; St. Luke´s College of Medicine, Quezon, 1112, Philippines; Goethe University Frankfurt, Frankfurt am Main, 60629, Germany; Buenos Aires University, Buenos Aires, C1035, Argentina; Fondazione Policlinico Universitario A. Gemelli, Rome, 00168, Italy; University of Birmingham, Birmingham, B15 2SQ, United Kingdom; GATOT Subroto Army Hospital, Jakarta, 10410, Indonesia; University of Geneva, Geneva, 1205, Switzerland; University of the Philippines, Manila, 1101, Philippines; College of Obstetrics and Gynecology, Singapore, 328836, Singapore; Catholic University of Leuven, Leuven, 3000, Belgium; Universidad Peruana Cayetano Heredia, Lima, 15102, Peru; University of Sydney, Sydney, 2050, Australia; Universidad Federal Fluminense Noterói, Rio de Janeiro, 24220-900, Brazil; University of Pittsburgh, Pittsburgh, 15260, USA; Hudson University, Clayton Victoria, 3168, Australia; Royal Thai College of Obstetricians and Gynecologists, Thai Medical Council Committee, Bangkok, 10310, Thailand; Banaras Hindu University, Varanasi, 221005, India; Aristotle University of Thessaloniki, Thessaloniki, 54124, Greece; State University of Rio de Janeiro (UERJ), Rio de Janeiro, 20551-030, Brazil; Oncology Committee of the American Association of Gynecological Laparoscopists (AAGL), Pune, 411004, India; University of Clermont-Ferrand, Clermont-Ferrand, 63001, France; University College London Hospitals, London, NW1 2BU, United Kingdom; Charité University, Berlin, 10117, Germany; Ege University, Izmir, 35040,Turkey; University of the Philippines, Manila, 72, Philippines; University of Nicosia, Egkomi, 2408, Cyprus; Radboud University, Nijmegen, 6525, Netherlands; University of Constanta, Constanta, 900573,Romania; Carl von Ossietzky University Oldenburg, 26131, Germany; University of Heidelberg, Heidelberg, 69117, Germany; Peking Union Medical College Hospital, Beijing, 100730, China; Special Interest Group Adhesions Research of the European Society for Gynaecological Endoscopy, Dubai, 12119, UAE

**Keywords:** Gynaecological surgery, tissue adhesions, prevention and control, health-planning recommendations

## Abstract

Adhesions are recognised as one of the most common complications of abdominal surgery; their diagnosis and prevention remains a significant unmet need in surgical therapy, affecting negatively a patient’s quality of life and healthcare budgets. In addition, postoperative pelvic adhesions pose a high risk of reduced fertility in women of childbearing age. These 2023 Global Recommendations on Adhesion Prevention in Gynaecological Laparoscopic Surgery provide agreed-upon statements to guide clinical practice, with the ultimate goal of improving patient outcomes.

## Introduction

Knowing the persistent significance of the burden caused by adhesions in open and in laparoscopic surgery, the necessity to regularly reformulate recommendations (De Wilde et al., 2022) for the current and present scenario is important to enhance women’s health (De Wilde et al., 2012). The latest recommendations were published as a global consensus of international universities and scientific societies. As tasks for the future, more prospective randomised trials, clinically validated risk scores, nomograms, registries and biobanks, implementing genetics and ethnicity, are needed. Scientifically evaluating studies should have different and individually documented clinical endpoints e.g. pain, pregnancy, abdominal discomfort, bowel obstruction, treatment readmission rate and recurrent surgery (Torres-de la Roche et al., 2019).

## Method

The following statements summarise the opinions of 34 international experts, scientific institutions and universities in the field of minimally invasive surgery and research on adhesion prophylaxis. They met to review the gaps in the evidence, the information given to surgeons during their training and key issues that should be emphasised in future research. The present global recommendations paper was developed through a process of consensus building. This involved the administration of a questionnaire to identify the key topics for discussion. This was followed by three rounds of discussion, with a final meeting in person. The aim of the recommendations is to highlight the main problems associated with this unwanted complication of gynaecological surgery and to alert surgeons to ways in which it could be reduced, and eventually prevented, to improve patient outcomes.

### 1. Surgery: Is surgery needed, then microsurgical

First consideration is the indication for surgery; no operation means no iatrogenic adhesiogenesis. When we still need to go through the process of surgery, it should be performed in a minimally traumatic fashion reducing tissue trauma as much as possible, using the microsurgical principles (Gomel and Koninckx, 2016; Torres- de la Roche, 2023).

### 2. Present: Respect for and gentle manipulation of the peritoneal tissue

The confirmed techniques of using thin, monofilament and non-absorbable sutures, avoiding powdered gloves, reducing electrocoagulation and thereby tissue ischemia induction, avoiding major bleeding and evacuating blood clots and minimising procedure time still remain in place (Hirschelmann et al., 2012). Full peritonealisation of foreign bodies e.g. meshes, or deep sutures in e. g. myomectomy is beneficial and should also be incorporated in adhesion reduction strategies (Toneman el al., 2023).

### 3. Diagnosis: Second-look laparoscopy remains the gold standard

Currently adhesions tend to be diagnosed when patients present with problems such as subfertility, bowel complaints or pain and this is usually in combination with surgical treatments. Non-invasive diagnostics like CINE-MRI and the sliding test during ultrasound may be the future; however, there is a paucity of validated studies regarding the sensitivity and specificity of these newer methods, as well as deficiencies in training programs to guarantee an appropriate learning curve for an accurate diagnosis. CT-scan is a valuable tool in diagnosing acute adhesion-related bowel obstruction because of its ability to accurately visualise the consequences of reduced bowel movement but it remains deficient when the latter is not significantly impaired e.g. in chronic pelvic pain or subfertility (De Wilde et al., 2022). The second-look laparoscopy, as an evaluating tool in adhesion formation and, at the same time, allowing concomitant adhesiolysis, remains the gold standard. However a thorough clinical assessment, including a detailed history, can elucidate potential adhesion-related disease (Torres-de la Roche et al., 2019).

### 4. Adhesion prophylactic agents: Known prophylactic agents are underused

Self- (Luo et al, 2020), and auto-cross-linked hyaluronic acid (Pellicano et al., 2003), gelified starch powder (Krämer et al, 2023), bioabsorbable membranes (Diamond et al., 2012) and hydroflotation (Trew et al., 2011), have all been shown to be effective as adhesion reducing agents in clinical settings; however, they are underused at present. Future considerations include medicated adhesion prophylactic agents and combining drugs with existing mechanical medical products. Prospective randomised trials in adhesiogenic disease or in patients with pre-existing adhesions will be of benefit. Expert opinion papers should be considered of high value to point out specific benefits of adhesion reduction measurements (Lundorff et al., 2015).

### 5. Adhesion risk: Drugs could be working on a metabolic level

When reviewing the patient’s complaints to assess the existence or severity of adhesions, visual analogue pain scales can be helpfully subjective, and bowel obstruction can realistically only be evaluated by a major registry. Most risk scores are not yet clinically validated (Lundorff et al., 2015), or still lack a bowel adhesion classification, like the CLAS-score (Lier et al., 2021). Surgeries especially prone to adhesion formation, invariably include endometriosis and oncological cases, although all surgeries can provoke adhesions (Torres-de la Roche et al., 2023). Repeat surgeries also enhance the relative adhesion induction risk. New drugs or technologies minimising the impact of such surgeries on a cellular or metabolic levels, e.g. alanyl-glutamine, could modify the healing process in the future, aiming at a goal of zero iatrogenic adhesion formation (Chizen et al., 2023). Antioxidants could also work at the same level (Koninckx et al., 2013; Mynbaev et al., 2002).

### 6. Microclimate: Microclimate is worth further exploration

The microclimate during laparoscopic surgery plays a crucial role and can be positively influenced by reducing the intraabdominal pressure, lowering the intraperitoneal temperature without reducing the whole body temperature, humidifying of the gas (Breuer et al., 2022), rinsing during the whole procedure but avoiding saline solution intraperitoneally, and eventually using molecular gases like O2 and N2O (Mynbaev et al., 2002). These factors should be further evaluated and classified before being implemented widely.

### 7. Individual surgeon’s standard: Awareness is key

Every surgeon should have an individual standard depending on the availability of surgical instrumentation, budget, qualification and local situation. Informed consent could enhance awareness on all sides (Torres-de la Roche et al., 2019).

## Conclusion

In light of the aforementioned statements, it can be concluded that efforts should be undertaken to produce GLOBAL CONSENSUS or GUIDELINES that are multidisciplinary and endorsed by scientific societies and universities. This is key to ensuring there is more awareness on all levels from the patient and surgeon to health authorities, insurance companies and decision- making governments. Furthermore, the burden of adhesions is not specific to gynaecology and it is imperative other medical disciplines with their specific needs and problems are involved.
